# School Absenteeism due to Toothache among Secondary School Students Aged 16–18 Years in the Ha'il Region of Saudi Arabia

**DOI:** 10.1155/2016/7058390

**Published:** 2016-02-18

**Authors:** Sameer Shaikh, Ammar Ahmed Siddiqui, Mohammad Aljanakh

**Affiliations:** ^1^Department of Oral Diagnosis & Oral Medicine, College of Dentistry, University of Ha'il, Ha'il 2440, Saudi Arabia; ^2^Department of Dental Public Health, College of Dentistry, University of Ha'il, Ha'il 2440, Saudi Arabia; ^3^Department of Restorative Dentistry, College of Dentistry, University of Ha'il, Ha'il 2440, Saudi Arabia

## Abstract

*Objective*. This study assessed the impact of toothache on school attendance among secondary school students in the Ha'il Region, Saudi Arabia.* Methods*. A cross-sectional, paper based survey was conducted among 16–18-year-old students of public sector secondary schools in the Ha'il Region, Saudi Arabia.* Results*. Of the 510 students selected from the participating schools, 480 were analyzed (94.1%). Of the sample, 50.4% were boys. Among the participants in the study, 86 students reported school absence due to toothache in the six months prior to the survey. Consequently, the prevalence of absenteeism due to toothache in this study was of 18%.* Conclusion*. The prevalence of school absenteeism due to toothache among students in the Ha'il Region was low. Yet, still, missed school days due to toothache may have implications for students also in the Ha'il Region, Saudi Arabia, as school absenteeism leads to missed opportunities for learning and academic advancement.

## 1. Introduction

Orodental problems affect people physically and psychologically and influence how they grow, enjoy life, look, speak, chew, taste food, and socialize, as well as their feelings of social well-being [1]. Severely decayed teeth have an important impact on individual's general health, nutrition, growth, and body weight by causing discomfort, pain, sleeping problems, learning disorders, and absence from school [2]. Limitations in functioning and performances of expected roles in school-aged adolescents aged 18 years or younger include problems with school activities such as inability to attend school and difficulties in learning in classroom as well as doing homework [3]. Earlier studies have reported that chronic illness can hinder the students' ability to prosper in school [4, 5]. In this perspective, dental problems were found to influence school performance and absenteeism [4, 6, 7]. There exists only very few reports on school absence specifically due to toothache in school-aged population [3].

To the best of our knowledge, no study thus far has analyzed the issue of school absenteeism due to toothache among school students in Saudi Arabia. Therefore, the aim of this cross-sectional study was to assess the impact of dental pain on school attendance among adolescents studying in secondary schools in the Ha'il Region, Saudi Arabia.

## 2. Materials and Methods

### 2.1. Research Design and Sampling

A cross-sectional, paper based survey was conducted among students of public sector secondary schools in the Ha'il Region, Saudi Arabia, from September 2015 to October 2015. Protocol was approved by the Ethics Committee of University of Ha'il. Permission for the current study was also obtained from the Department of Dentistry, Ministry of Health, Saudi Arabia. A two-stage sampling procedure was adopted to select the sample. In the first stage, the public sector secondary schools in the city were divided into four sections according to the geographical location: north, east, west, and south. Two schools (one for females and the other for males) were randomly selected from each section to get a sample of eight schools. In the second stage, a convenience sample of 170 students from each age group with approximately equal gender distribution was collected for the study. A day was set for each school to collect the data. The schools administration had been informed before the visit to schools and permission was obtained. Parents' approval and the participants' informed consent were acquired at the study onset.

### 2.2. Data Collection

Data on absence were collected from daily school attendance records for 6 months. A specially designed form was used specifying reasons for absence: dental (dental appointments for toothache, dental pain without visit to the dentist), medical (medical appointment and medical illnesses), or social reasons (such as attending relatives' weddings, funerals, or helping parents with housework or going on holidays with parents).

### 2.3. Data Analysis

Statistical analysis was performed using SPSS version 20. The differences in school absence due to toothache among genders of various age groups were investigated using chi-square test. *P* values ≤ 0.05 were considered statistically significant.

## 3. Results

**Figure 1:**
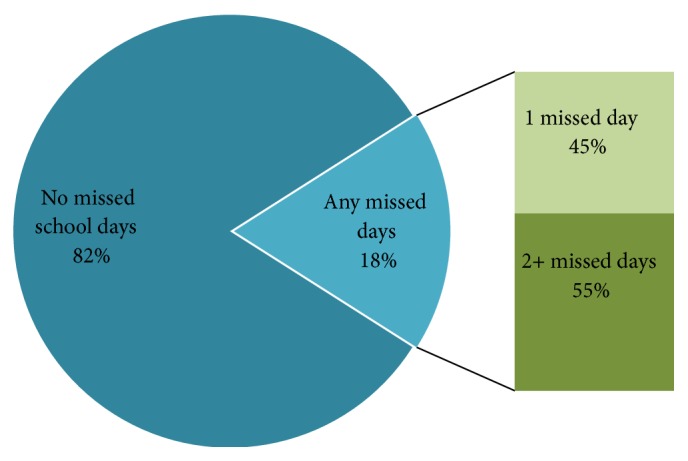
Percentage of secondary school students with school absences due to toothache in the Ha'il Region of Saudi Arabia.

**Figure 2:**
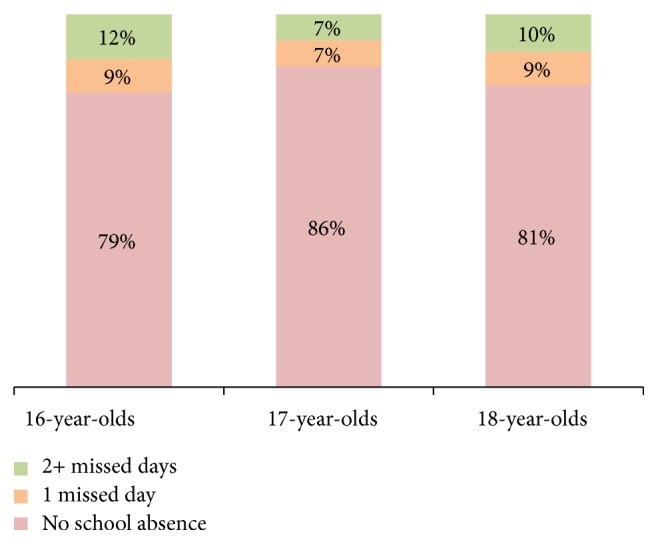
Age-wise percentage of secondary school students with missed school days due to toothache in the Ha'il Region of Saudi Arabia.

Of the 510 selected students, 480 were analyzed (94.1%). Male students comprised 50.4% of the study participants. Distribution of study participants by age, gender, and school class is presented in Table 1. Of the 480 students in our sample, 394 (82%) had not missed any school days due to toothache during the past 6 months. Among the participants in the study, 86 students reported school absence due to toothache in the six months prior to the survey. Consequently, the prevalence of absenteeism due to toothache in this study was of 18%. The majority of these students (55%, *n* = 47) report missing two or more days of school due to toothache (including 31% with two, 11% with three, and 13% with more than three days), while the remaining 45% (*n* = 39) report missing one day of school (Figure 1).  Table 2 illustrates the numbers of school days missed due to toothache. The number of days absent in 16-year-old students (21%, *n* = 34) was higher than in 17- (14%, *n* = 22) and 18-year-old (19%, *n* = 30) students (Figure 2). The association between school absenteeism and gender indicated that the prevalence of absenteeism resulting from toothache was higher for 16- and 18-year-old female students (*P* ≤ 0.05) (Table 3).

## 4. Discussion

Ha'il Region is located in the north of Saudi Arabia. According to the survey conducted in 2013 by the Central Statistics Department, Ministry of Economy and Planning, Saudi Arabia, there were 46 government-run secondary schools in the Ha'il Region, 22 for males and 24 for females [8]. For sampling purposes, the public sector secondary schools in Ha'il Region were divided into four sections according to the geographical location: north, east, west, and south. Two schools (one for females and the other for males) were randomly selected from each section to get a sample of eight schools. In Saudi Arabia, secondary education lasts for three years after intermediate education, for the ages of 14–18 [9].

Inferior oral health has been accepted as having adverse effects on day-to-day performance and quality of life. Students with poorer oral health status were more likely to experience dental pain, miss school, and perform poorly in academic activities. The problem of school absence is critical, as it can affect students' quality of life in terms of missed academic learning. In addition, school absenteeism is likely to be correlated with missed days of work for parents who have to attend to the children suffering from toothache or take them for treatment. School-aged children and adolescents with poor general health are more likely to have inferior academic performance and spiraling school absence. However, the relationship between students' school performance and their oral health problems has rarely been explored in developing countries [7, 10, 11].

The present study's findings were consistent with few of the previous studies. In Thailand, 22.5% percent of students reported school absence for any dental reason during the 1-year observation period [12].

In another study from Thailand, over a 3-month period, nearly 10% of students absented themselves from school due to toothache [3]. Within the past 12 months, 21.8% of all US children and adolescents aged 6–17 years had dental problems, such as toothache, decayed teeth, or unfilled cavities. The mean number of days of school absence was significantly higher among students with an unmet therapeutic dental need in the presence of a dental problem compared to those with no unmet dental need [13].

On the contrary, in North Carolina, 96.1% students had not missed any school days as a result of dental pain or infection during the past 12 months [7].

The results of this study are limited by the small sample size. A larger sample size would achieve more comprehensive results. On the research front, it is necessary to identify other orodental ailments associated with school absence. Moreover, it is also important to assess the impact of orodental conditions on the academic performance, since students experiencing toothache are more likely to be distracted and unable to concentrate on schoolwork. In addition, chewing difficulties due to dental problems often lead to limited choice of foods and poor nutrition with further impact on school performance. As a suggestion, establishment of school-based dental clinics may lead to the improvement of oral health of the students. Ultimately, this improvement may act as a vehicle to improve their educational experience.

## 5. Conclusion

Although a relatively small percentage (approximately 18%) of our sample missed school as a result of dental pain, still it is important to consider the adverseness of orodental problems on school attendance along with academic performance. These findings accentuate the probability that school absence is not a stand-alone aspect in considerations of school performance, providing further evidence that students experiencing pain or infection may have a diminished educational experience because their discomfort may inhibit their ability to perform well while at school. Like their international counterparts, missed school days due to toothache may have implications for school students also in the Ha'il Region, Saudi Arabia, as school absenteeism leads to missed opportunities for learning and academic advancement.

## Figures and Tables

**Table 1:** Distribution of study participants by age, gender, and school class.

Variables	Total = 480 (100%)	Female = 238 (49.6%)	Male = 242 (50.4%)
	*N* (%)	*N* (%)	*N* (%)
Age (years)			
16	163 (34%)	80 (33.6%)	83 (34.3%)
17	158 (32.9%)	78 (32.8%)	80 (33.1%)
18	159 (33.1%)	80 (33.6%)	79 (32.6%)
School class			
10	163 (34%)	77 (32.4%)	86 (35.5%)
11	156 (32.5%)	83 (34.9%)	73 (30.2%)
12	161 (33.5%)	78 (32.8%)	83 (34.3%)

**Table 2:** Numbers of school days missed due to toothache among secondary school students in the Ha'il Region, Saudi Arabia.

Age groups	Absent for 1 day	Absent for 2 days	Absent for 3 days	Absent for >3 days
16 (*n* = 163)	14 (8.5 %)	13 (7.9%)	3 (1.8%)	4 (2.4%)
17 (*n* = 158)	11 (6.9%)	6 (3.7%)	3 (1.8%)	2 (1.2%)
18 (*n* = 159)	14 (8.8%)	8 (5.0%)	3 (1.8%)	5 (3.1%)
Total (*n* = 480)	39 (8.1%)	27 (5.6%)	9 (1.9%)	11 (2.3%)

**Table 3:** Age- and gender-wise distribution of study participants with school absences due to toothache in the Ha'il Region, Saudi Arabia.

Age	Gender	School days missed due to toothache	*P* value
		*n* (%)	
16 (*n* = 163)	Female	23 (14.1%)	0.01^*∗∗*^
	Male	11 (6.7%)	

17 (*n* = 158)	Female	8 (5.1%)	0.18
	Male	14 (8.9%)	

18 (*n* = 159)	Female	22 (13.8%)	0.05^*∗*^
	Male	8 (5%)	

^*∗*,*∗∗*^ *P* ≤ 0.05.
